# Cognitive and neuropsychiatric endophenotypes in amyotrophic lateral sclerosis

**DOI:** 10.1093/braincomms/fcad166

**Published:** 2023-05-19

**Authors:** Emmet Costello, Marie Ryan, Bronagh Donohoe, Caoimhe Kavanagh, Marta Pinto-Grau, Mark Doherty, Russell Lewis McLaughlin, Caroline McHutchison, Sharon Abrahams, Mark Heverin, Orla Hardiman, Niall Pender

**Affiliations:** Academic Unit of Neurology, Trinity Biomedical Science Institute, Dublin D02 R590, Ireland; Psychology Department, Beaumont Hospital, Dublin D09 V2N0, Ireland; Academic Unit of Neurology, Trinity Biomedical Science Institute, Dublin D02 R590, Ireland; Academic Unit of Neurology, Trinity Biomedical Science Institute, Dublin D02 R590, Ireland; Academic Unit of Neurology, Trinity Biomedical Science Institute, Dublin D02 R590, Ireland; Academic Unit of Neurology, Trinity Biomedical Science Institute, Dublin D02 R590, Ireland; Psychology Department, Beaumont Hospital, Dublin D09 V2N0, Ireland; Academic Unit of Neurology, Trinity Biomedical Science Institute, Dublin D02 R590, Ireland; Academic Unit of Neurology, Trinity Biomedical Science Institute, Dublin D02 R590, Ireland; Human Cognitive Neurosciences–Psychology, School of Philosophy, Psychology, Language Sciences, The University of Edinburgh, Edinburgh EH8 9AD, UK; Euan MacDonald Centre for Motor Neuron Disease Research, The University of Edinburgh, Edinburgh EH16 4SB, UK; Human Cognitive Neurosciences–Psychology, School of Philosophy, Psychology, Language Sciences, The University of Edinburgh, Edinburgh EH8 9AD, UK; Euan MacDonald Centre for Motor Neuron Disease Research, The University of Edinburgh, Edinburgh EH16 4SB, UK; Academic Unit of Neurology, Trinity Biomedical Science Institute, Dublin D02 R590, Ireland; Academic Unit of Neurology, Trinity Biomedical Science Institute, Dublin D02 R590, Ireland; Psychology Department, Beaumont Hospital, Dublin D09 V2N0, Ireland

**Keywords:** endophenotype, amyotrophic lateral sclerosis, cognition

## Abstract

First- and second-degree relatives of people with amyotrophic lateral sclerosis report higher rates of neuropsychiatric disorders, indicating that risk genes may be pleiotropic, causing multiple phenotypes within kindreds. Such phenotypes may constitute a disease endophenotype that associates with disease liability. We have directly investigated cognitive functioning and neuropsychiatric traits among relatives of people with amyotrophic lateral sclerosis to identify potential endophenotypes of the disease. In a family-based, cross-sectional study design, first- and second-degree relatives of people with amyotrophic lateral sclerosis (*n* = 149) were compared to controls (*n* = 60) using an in-depth neuropsychological and neuropsychiatric assessment. Subgroup analyses examined the effect of family history and *C9orf72* repeat expansion status (*n* = 16 positive carriers). Relatives of people with amyotrophic lateral sclerosis had lower scores on executive functioning, language and memory tasks compared to controls, with large effect sizes observed on object naming (*d* = 0.91, *P* = 0.00001) and phonemic verbal fluency (*d* = 0.81, *P* = 0.0003). Relatives also had higher autism quotient attention to detail traits (*d* = −0.52, *P* = 0.005), lower conscientiousness (*d* = 0.57, *P* = 0.003) and lower openness to experience personality traits (*d* = 0.54, *P* = 0.01) than controls. These effects were typically larger in relatives of people with familial, rather than sporadic, amyotrophic lateral sclerosis and were present in both gene carrier and non-carrier relatives of probands with a *C9orf72* repeat expansion. Poorer phonemic fluency and object naming, along with autism and personality traits, are more frequent in relatives of people with amyotrophic lateral sclerosis. Among kindreds carrying the *C9orf72* repeat expansion, these traits were identified in relatives regardless of their carrier status, suggesting the presence of a disease-associated endophenotype that is not exclusively mediated by the *C9orf72* expansion.

## Teaser

Costello *et al*. describe deep phenotyping of first- and second-degree relatives of people with amyotrophic lateral sclerosis. They report that relatives of people with amyotrophic lateral sclerosis show a distinct cognitive and neuropsychiatric profile which may constitute a disease endophenotype.

## Graphical Abstract

**Graphical abstract:**
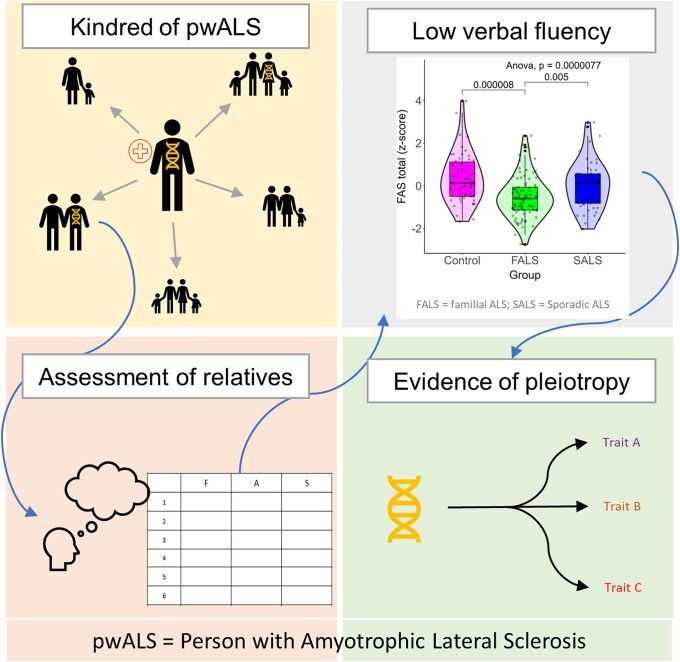

## Introduction

Amyotrophic lateral sclerosis (ALS) is a neurodegenerative condition characterized by the degeneration of upper and lower motor neurons, leading to progressive paralysis and death.^1^ Extra-motor pathology is also commonly observed in frontal, prefrontal and temporal regions^2^ and is most commonly associated with executive and behavioural dysfunction, with 14% of people with ALS (pwALS) meeting criteria for co-morbid frontotemporal dementia (FTD)^3^ and an additional 35% showing milder cognitive/behavioural change. Meta-analysis shows that there is now strong evidence for deficits in language, verbal fluency, executive functioning, social cognition and memory in a large portion of pwALS,^4^ indicating that ALS and FTD represent two aspects of a disease spectrum, known as ALS frontotemporal spectrum disorder (FTSD).^5^

The most common genetic association with ALS and FTD in populations of European extraction is the presence of a hexanucleotide repeat expansion in *C9orf72.*^6^ In Ireland, the *C9orf72* repeat expansion is observed in ∼41% of familial ALS cases and ∼5% of apparently sporadic ALS cases.^7^ The presence of the *C9orf72* expansion is associated with earlier disease onset, a higher likelihood of cognitive and behavioural impairment, non-motor cortex changes, a family history of neurodegenerative diseases with incompletely penetrant inheritance and reduced survival.^8^ Notwithstanding the discovery of the *C9orf72* repeat expansion and other variants, the genetic cause of ∼50% of familial ALS in Ireland and ∼90% of sporadic ALS cases remains unknown.

Family aggregation studies have demonstrated higher rates of schizophrenia, psychotic illness, suicide, autism, obsessive compulsive disorder (OCD) and alcoholism among first- and second-degree relatives of pwALS.^7,9^ This association is supported by genomic data that indicate a 14% overlap in genetic susceptibility to ALS and schizophrenia.^10^ Furthermore, the presence of specific neuropsychiatric illnesses in family members is predictive of the cognitive and behavioural phenotype of the proband with ALS, e.g. a family history of neurotic disorders is associated with language dysfunction in pwALS.^11^ Thus, growing evidence suggests that ALS risk genes may be pleiotropic (having multiple phenotypic effects) and that neuropsychiatric characteristics among ALS kindreds may represent disease endophenotypes that require additional exploration.

Endophenotypes are quantitative traits that correlate with disease liability.^12^ Endophenotypes are measurable in both affected and unaffected individuals, can help identify individuals at risk prior to the disease onset and provide greater statistical power in localizing and identifying disease-related genes than dichotomizing between affected and unaffected status alone.^13^ For example, abnormal smooth pursuit eye tracking is observed in 40–80% of people with schizophrenia, 25–45% of their first-degree relatives but less than 10% of healthy controls.^12^ While the pathophysiology of abnormal eye tracking is complex, it serves as a useful endophenotype as it appears to segregate with risk of illness in the population, can be reliably measured, is heritable, state-independent and is present at a higher rate in unaffected family members than in the general population.

The aim of this study was to carry out deep phenotyping of apparently unaffected relatives of pwALS (i.e. individuals with one or more family members with ALS but no clinical or motor symptoms) to characterize cognitive functioning and neuropsychiatric traits. We examined the effect of family history (i.e. familial ALS versus sporadic ALS) and *C9orf72* gene status on these outcomes. We hypothesized that relatives with a stronger family history of ALS and those who were *C9orf72* positive would be more likely to display poorer cognitive performance and abnormal neuropsychiatric traits.

## Materials and methods

### Design

This study utilized a quantitative, observational, cross-sectional and family-based research design. First- and second-degree relatives of pwALS were compared to healthy controls on a battery of neuropsychological and neuropsychiatric measures.

### Participants

PwALS were identified through the Irish ALS register.^14^ First- and second-degree relatives were recruited via chain referral sampling whereby pwALS provided contact information for interested family members. With the pwALS consent, first- and second-degree relatives were contacted and informed about the research project. Healthy controls were recruited through established recruitment networks, such as local advertising and community groups. Individuals completed the assessment in their home, in Beaumont Hospital or Trinity Biomedical Sciences Institute, Dublin, Ireland.

Inclusion criteria for relatives of pwALS included (i) a first- or second-degree relative of a person with ALS; (ii) age of over 18 years; and (iii) a native English speaker. Exclusion criteria for all participants were (i) a history of neurological conditions that affect cognition, e.g. stroke, traumatic brain injury, severe epilepsy, etc.; (ii) an established history of a learning disability or developmental disorder; (iii) active alcohol dependence syndrome; (iv) severe and active mental illness; and (v) current use of major neuroleptic or psychoactive medication. Healthy controls were also excluded if they had a family history of ALS or FTD.

### Materials

A semi-structured interview was undertaken to capture demographic and clinical information. Demographic details included the participants’ date of birth, gender, education (years in formal education and highest qualification), occupation and marital status. Clinical details included medication; alcohol intake (measured in units per week); history of depression (and the severity); history of diabetes mellitus, hypertension and hypercholesterolaemia; history of head trauma or exposure to heavy metals; and history of intellectual or learning disability.

The neuropsychological assessment comprised an extensive battery of cognitive tests (see Table 1). This assessment examined five key cognitive domains, including intellectual functioning and those commonly affected in pwALS,^4^ such as executive functions, language, memory and social cognition.

**Table 1:** Summary of the tests used in the neuropsychological battery and the cognitive function they measure

Neuropsychological test		Cognitive function assessed
**Intellectual functioning**		
Test of Premorbid Functioning UK (TOPF-UK)^15^		Premorbid intellectual functioning
Wechsler Abbreviated Scale of Intelligence 2nd edition (WASI-II)^15^	Vocabulary	Word (semantic) knowledge
	Matrix reasoning	Non-verbal abstract problem-solving and inductive reasoning
**Executive functioning**		
Verbal fluency	FAS test^16^	Phonemic and semantic fluency with unrestricted and restricted conditions
	Animal fluency^17^	
Colour Word Interference Task (CWIT)^18^	Colour naming	Inhibitory control, error monitoring, selective attention and cognitive flexibility
	Word reading	
	Inhibition	
	Inhibition/switching	
Wechsler Adult Intelligence Scale—4th edition (WAIS-IV)^19^	Digit span forwards	Attention and working memory
	Digit span backwards	
	Digit span sequential	
Iowa Gambling Task (IGT)^20^		Emotion-based decision-making
**Language**		
Boston Naming Task (BNT)^21^		Confrontational naming
**Memory**		
Rey Auditory Verbal Learning Test (RAVLT)^22^		Encoding, learning recall and recognition of verbal information
Wechsler Memory Scale—3rd edition (WMS-III)^23^	Logical memory I and II	Encoding, recall and recognition of verbal information
Rey Complex Figure Test (RCFT)^24^		Visuospatial and constructive ability and visuospatial memory
**Social cognition**		
Reading the Mind in the Eyes Task (RMET)^25^		Theory of mind: perception, recognition and naming of facial (eye region) expressions of emotions and thoughts

To examine neuropsychiatric traits, participants completed a comprehensive series of neuropsychiatric questionnaires. These questionnaires assessed the presence and severity of symptoms associated with depression, anxiety, obsessive compulsive disorder (OCD), impulsiveness, apathy, autism, attention deficit hyperactivity disorder (ADHD), psychosis and personality traits (see Table 2). The neuropsychiatric questionnaires were primarily administered online, using the survey platform Qualtrics. A paper version alternative was provided if the person was unable to use a computer or declined the online version.

**Table 2:** Neuropsychiatric questionnaires administered

Neuropsychiatric questionnaire	Psychiatric trait/symptom/behaviour assessed
Patient Health Questionnaire—9 (PHQ-9)^26^	Symptoms and severity of depression
Generalized Anxiety Disorder—7 (GAD-7)^27^	Symptoms and severity of anxiety
Obsessive Compulsive Inventory Revised (OCI-R)^28^	Symptoms relating to obsessions and compulsions including washing, checking, ordering, obsessing, hoarding and neutralizing
Barrett Impulsiveness Scale (BIS-11)^29^	Symptoms of impulsive behaviours and preferences in five domains: attention, cognitive stability, perseverance, self-control and cognitive complexity
Dimensional Apathy Scale (DAS) ^30^	Apathy in three different dimensions: executive, emotional and cognitive/behavioural initiation
Autism spectrum quotient (AQ)^31^	Social and non-social aspects of behavioural and cognitive difficulties associated with autism
Adult ADHD Self-Report Scale (ASRS)^32^	Symptoms of ADD/ADHD based on the DSM-IV criteria
Community Assessment of Psychic Experiences (CAPE-P15)^33^	Positive symptoms of psychosis: persecutory ideation, bizarre experiences and perceptual abnormalities
Ten-Item Personality Inventory (TIPI)^34^	Five main personality traits: extroversion, openness, agreeableness, neuroticism and conscientiousness

### Procedure

Neuropsychological assessment took between 2 and 3 hours to complete, with short breaks to minimize fatigue. Participants were later emailed with a link to the online neuropsychiatric traits questionnaire. For participants who did not wish to complete the questionnaire online, a paper version was provided and returned by mail.

All participants provided a blood sample for DNA extraction and analysis. All samples were given a unique code at source and stored in the Trinity Biomedical Sciences Institute, Dublin, Ireland. DNA samples were tested in-house for the presence of the pathogenic *C9orf72* repeat expansion. Participants with 30 or more hexanucleotide repeats were deemed positive for *C9orf72*.^35^ Expansions with 20–29 repeats were considered intermediate *C9orf72* carriers. Participants with 19 or less hexanucleotide repeats were deemed *C9orf72* negative.

Participants and researchers were not informed of the genetic test results of any individual, and the geneticists performing the genetic tests did not have access to participant identifiers. Genetic status was added to the database by the ALS research manager M.H., and the ID codes were then removed to anonymize for analysis. This ensured that all relevant parties were blinded to the participants’ genetic status throughout the study.

### Ethical considerations

Participant consent was obtained according to the Declaration of Helsinki, and ethical approval was granted by the Beaumont Hospital Research Ethics Committee (REC Reference 15/40). Informed written consent was obtained from all participants.

### Statistical analysis

All analyses were carried out using R statistical software, version 3.6.3.^36^ The R code used to carry out analyses can be found at https://github.com/emmetcostello/Endophenotypes-in-ALS. The following R packages were used: tidyverse, ggplot2, readxl, here, summarytools, rstatix, ggpubr, effectsize, pastecs, car, scales, ggforce, ggpubr, knitr, kableExtra, table1, MatchIt, QuantPsyc, factoextra, NbClust, GGally and plotly.

G*Power software (version 3.1)^37^ was used to calculate the minimum sample size required for this study. An alpha value of *P* = 0.05 was adopted, and a desired power of 0.8 was specified. Based on pre-symptomatic studies of ALS gene carriers,^38,39^ medium to large effect sizes were expected when comparing unaffected relatives of pwALS to controls on cognitive outcomes (expected *d* = 0.7). Based on family-based studies of neuropsychiatric disorders, slightly smaller and more variable effect sizes were expected when comparing unaffected relatives of pwALS to controls on neuropsychiatric outcomes (expected *d* = 0.63).^40,41^ To carry out a one-way ANOVA, with an estimated effect size of *d* = 0.7, an alpha of *P* = 0.05 and a desired power of 0.8, a minimum sample size of 139 is required. When the estimated effect size was 0.63, the minimum sample needed was 170.

Two-sided Welch’s *t*-tests or Wilcoxon rank tests were carried out to compare neuropsychological performance in unaffected relatives of pwALS and healthy controls. One-way ANOVA or Kruskal–Wallis tests were undertaken to examine the effect of family history, comparing relatives of people with familial ALS (as defined by Byrne *et al*.^42^), relatives of people with sporadic ALS, and controls. *Post hoc* comparisons for significant main effects were carried out using Bonferroni–Holm tests. *C9orf72* positive gene carriers were compared to *C9orf72* negative gene carriers using Welch’s *t*-tests or Wilcoxon rank tests.

The Bonferroni–Holm correction was used to control for multiple comparisons (see supplementary Table 1 for corrected alphas). To control for the confounding effect of age, raw scores were converted to *Z*-scores using age-specific normative control data in test manuals or published articles. As normative data were not available for many of the neuropsychiatric questionnaires, multiple linear regressions were carried out to compare the neuropsychiatric traits of relatives and controls, holding age constant. Age and group status (i.e. relative or control) were entered as predictors, and each neuropsychiatric score was entered as outcome variables.

## Results

### Participant characteristics

Eighty-four pwALS were contacted, of whom 59 (70%) consented to having their family members approached to take part to the study. Out of 295 first- or second-degree relatives contacted, 240 (81%) participated to some degree, with 149 (62%) completing the full study (i.e. gave a blood sample, completed the neuropsychological assessment and the online neuropsychiatric questionnaire). Only those with complete data were included in this study.

One hundred and twenty-nine participants were a first-degree relative, and 20 were a second-degree relative of a pwALS. In total, 48 ALS kindreds were included in the analysis, and the median number of family members from each family was 3 (ranging from 1 to 13). Of 104 healthy controls contacted from the general population, 60 (58%) took part.

Participant demographic and clinical characteristics are outlined in Table 3. ALS relatives and controls were well matched in respect of gender, handedness, education, marital status and alcohol intake. However, the control group was older than the relatives’ cohort, with more controls in retirement. This age discrepancy was controlled for using age-stratified normative data and multiple linear regression (see the Statistical analysis section). Eleven families (*n* = 44 relatives) had at least one person with an intermediate or positive *C9orf72* expansion status.

**Table 3:** Participant demographic and clinical characteristics

	Relatives (*n* = 149)	Controls (*n* = 60)
**Sex**		
Female, *n* (%)	82 (55.0%)	32 (53.3%)
Male, *n* (%)	67 (45.0%)	28 (46.7%)
**Age**		
Age in years, mean (SD)	46.1 (17.4)	63.7 (10.2)
**Handedness**		
Left, *n* (%)	18 (12.1%)	8 (13.3%)
Right, *n* (%)	129 (86.6%)	52 (86.7%)
Ambidextrous, *n* (%)	2 (1.3%)	0 (0%)
**Education**		
Years in education, mean (SD)	16.7 (3.19)	16.4 (3.70)
**Highest education level**		
Apprenticeship, *n* (%)	7 (4.7%)	1 (1.7%)
Primary, *n* (%)	3 (2.0%)	1 (1.7%)
Secondary, *n* (%)	36 (24.2%)	19 (31.7%)
Tertiary, *n* (%)	103 (69.1%)	39 (65.0%)
**Marital status**		
Divorced, *n* (%)	2 (1.3%)	4 (6.7%)
Married, *n* (%)	82 (55.0%)	37 (61.7%)
Single, *n* (%)	57 (38.3%)	11 (18.3%)
Widowed, *n* (%)	8 (5.4%)	8 (13.3%)
**Familial versus sporadic family history**		
FALS, *n* (%)	91 (61.1%)	—
SALS, *n* (%)	58 (38.9%)	—
***C9orf72* repeat expansion status**		
Intermediate, *n* (%)	6 (4.0%)	—
Negative, *n* (%)	123 (82.6%)	—
Positive, *n* (%)	10 (6.7%)	—
Missing, *n* (%)	10 (6.7%)	—

FALS = familial ALS; SALS = sporadic ALS. Individuals with >30 repeats were deemed positive; 20–29 repeats were deemed intermediate.

### Cognitive functioning in relatives of pwALS

Welch’s *t*-tests and Wilcoxon rank tests found that relatives of pwALS performed significantly worse than controls on numerous tasks, across multiple cognitive domains (see Table 4). On executive tasks, relatives had poorer phonemic verbal fluency, made a greater number of inhibition errors and had a shorter backwards digit span than controls. On tests of language, relatives of pwALS had poorer object naming than controls. In terms of memory performance, relatives had poorer immediate list learning, poorer delayed story recall and poorer visuospatial reconstruction than controls. Initially, significant differences were observed in intelligence quotient (IQ) and the Iowa Gambling Task scores; however, these did not survive correction for multiple comparisons. A *post hoc* multiple regression analysis determined whether IQ differences could account for the deficits observed on language, executive functioning and memory (see supplementary Table 3). Significant differences between groups were maintained on verbal fluency, object naming and backwards digit span after controlling for IQ.

**Table 4:** Comparison of standardized neuropsychological performance of relatives of pwALS and control

		Relatives (*n* = 149)	Controls (*n* = 60)	*t/W* (df)	*P*	*d/r*
*Intellectual functioning*						
TOPF-UK	*FSIQ*	104.49 (12.2)	110.22 (12.37)	3.02 (106)	0.003	0.59
WASI-II	*FSIQ-2*	98.92 (14.11)	105.26 (16.12)	2.62 (94)	0.01	0.54
*Executive functioning*						
Verbal fluency	*FAS total z*	−0.33 (1.08)	0.36 (1.16)	3.79 (89)	0.0003*	0.81
	*Animals total z*	−0.13 (0.95)	−0.03 (1.15)	0.58 (94)	0.56	0.12
Colour word interference test	*Inhibition errors ss*	10.29 (2.61)	11.25 (1.67)	3.08 (158)	0.002*	0.49
	*Inhibition time ss*	10 (2.95)	10.68 (2.49)	1.66 (120)	0.10	0.3
	*Switching errors ss*	10.23 (2.33)	10.49 (2.23)	0.73 (106)	0.47	0.14
	*Switching time ss*	9.96 (2.85)	10.72 (2.74)	1.76 (106)	0.08	0.34
Digit span	*Forwards z* ^a^	0.14 (0.96)	0.38 (0.91)	4735	0.51	0.06
	*Backwards z*	0.08 (1.02)	0.72 (0.97)	4.19 (109)	0.00006*	0.8
	*Sequential z*	0.34 (0.98)	0.43 (0.93)	0.56 (115)	0.57	0.1
Iowa Gambling Task	*Total t*	45.98 (8.8)	49.95 (7.89)	2.36 (92)	0.02	0.49
	*Block 1 t*	46.98 (10.35)	44.98 (10.3)	−0.97 (87)	0.34	−0.21
	*Block 2 t*	49.56 (7.49)	50.56 (7.67)	0.7 (85)	0.48	0.15
	*Block 3 t*	46.92 (10.38)	49.29 (10.23)	1.14 (87)	0.26	0.24
	*Block 4 t*	46.03 (10.46)	50 (12.39)	1.68 (77)	0.1	0.38
	*Block 5 t*	42.87 (14.17)	50.27 (11.04)	2.93 (97)	0.004	0.6
*Language*						
BNT	*Spontaneous z*	−1.12 (1.42)	−0.19 (0.66)	6.42 (201)	<0.00001*	0.91
	*Cued z*	−1.79 (2.06)	−0.53 (0.94)	6.01 (201)	<0.00001*	0.85
*Memory*						
RAVLT	*Immediate z*	0.4 (1.23)	1.08 (1.35)	3.31 (98)	0.001*	0.67
	*Delayed z*	0.11 (1.17)	0.65 (1.46)	2.52 (88)	0.01	0.54
	*Recognition z* ^a^	0.69 (1.09)	0.7 (0.9)	3661	0.38	0.06
Logical Memory	*Immediate z*	0.35 (0.95)	0.18 (0.89)	−1.17 (108)	0.25	−0.22
	*Delayed z*	0.13 (1.05)	0.73 (1.09)	3.48 (98)	0.0007*	0.7
	*Recognition raw*	25.55 (2.75)	26 (2.25)	1.2 (128)	0.23	0.21
RCFT	*Copy z* ^a^	0.42 (1.69)	0.22 (1.48)	4003.5	0.94	0.005
	*Copy time z*	−0.51 (1.07)	−0.96 (0.73)	−3.36 (144)	0.001	−0.56
	*Immediate z*	−0.1 (1.53)	0.91 (1.61)	3.99 (91)	0.0001*	0.84
	*Delayed z* ^a^	−0.19 (2.42)	0.63 (1.51)	4843	0.005	0.2
	*Recognition z*	−0.35 (1.4)	0.25 (1.19)	2.85 (118)	0.005	0.52
*Social cognition*						
RMET	*Total z*	−0.19 (1.08)	−0.17 (0.87)	0.13 (136)	0.9	0.02

For parametric data, scores are reported as means (standard deviations), Welch’s *t*-value (degrees of freedom), *P*-value and Cohens *D*.

a Indicates data were not normally distributed and that scores are represented using medians (standard deviations), Wilcoxon rank *W*, *P*-value and Spearman’s *r*. All scores (except logical memory recognition) are standardized using age-specific norms and presented as *IQ*, *Z-*, *t- or scaled scores*, with lower scores indicating worse performance.

* Indicates a significant difference after controlling for multiple comparisons using Bonferroni–Holm method.

### Effect of family history

**Figure 1:**
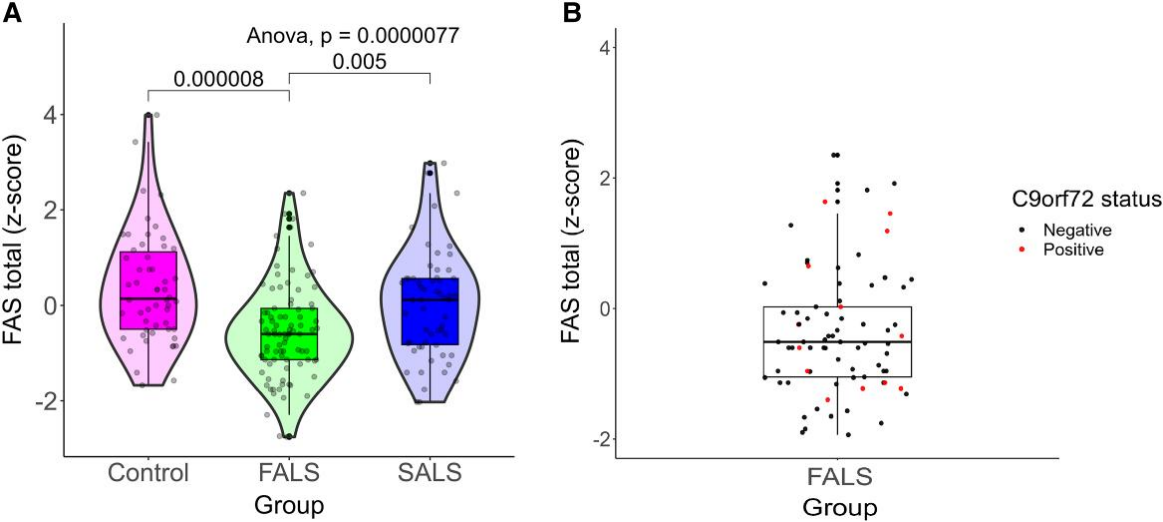
**Verbal fluency performance in relatives of people with familial ALS, sporadic ALS and controls.** (**A**) Violin plot of distribution of FAS verbal fluency *Z*-scores (each dot representing an individual data point) in FALS relatives (green), SALS relatives (blue) and controls (magenta). Significant one-way ANOVA main effect, *F*(2198) = 12.51, *P* = 0.0000077. *Post hoc* Tukey’s test significant between FALS relatives and controls, *P* = 0.000008, and between SALS relatives and FALS relatives, *P* = 0.005. (**B**) FAS verbal fluency *Z*-score data points in C9orf72 gene carrier (red) and non-C9orf72 carrier (black) relatives of people with FALS. FALS = familial amyotrophic lateral sclerosis; SALS = sporadic amyotrophic lateral sclerosis.

One-way ANOVA’s and Kruskal–Wallis tests found significant main effects for family history of ALS on phonemic verbal fluency, CWIT inhibition errors, backwards digit span, object naming, immediate list learning, delayed story recall and immediate visuospatial functioning (see Table 5). Mean cognitive performance was generally lowest in relatives of people with FALS, followed by SALS relatives and then controls. The effects of family history were most evident in phonemic verbal fluency performance (see Fig. 1A), where *post hoc* Bonferroni–Holm tests found that FALS relatives scored significantly worse than both controls and SALS relatives. In families of probands carrying the *C9orf72* repeat expansion, this observation was true for all relatives regardless of C9 repeat expansion carrier status (see Fig. 1B).

**Table 5:** Neuropsychological performance in FALS relatives, SALS relatives and controls

		FALS (*n* = 96)	SALS (*n* = 53)	Controls (*n* = 60)	*F/H*	*Post hoc* comparisons	
						Subgroups	*P*
*Executive functioning*							
Verbal fluency	*FAS total z*	−0.23 (0.85)	0.03 (1.07)	−0.03 (1.15)	12.51***	FALS versus HC	<0.001
						SALS versus HC	0.08
						FALS versus SALS	0.005
CWIT	*Inhibition errors ss*	10.43 (2.22)	10.07 (3.12)	11.25 (1.67)	3.69*	FALS versus HC	0.07
						SALS versus HC	0.03
						FALS versus SALS	0.38
Digit span	*Backwards z*	−0.01 (1.02)	0.21 (1.01)	0.72 (0.97)	9.31***	FALS versus HC	<0.001
						SALS versus HC	0.01
						FALS versus SALS	0.2
*Language*							
BNT	*Spontaneous z* ^a^	−0.7 (1.46)	−1.05 (1.35)	−0.12 (0.66)	28.75***	FALS versus HC	<0.05
						SALS versus HC	<0.05
						FALS versus SALS	>0.05
*Memory*							
RAVLT	*Immediate z*	0.38 (1.22)	0.44 (1.26)	1.08 (1.35)	5.93**	FALS versus HC	0.004
						SALS versus HC	0.01
						FALS versus SALS	0.77
Logical memory	*Delayed z*	0.84 (0.15)	0.1 (1.21)	0.73 (1.09)	6.29*	FALS versus HC	0.003
						SALS versus HC	0.003
						FALS versus SALS	0.79
RCFT	*Immediate z*	0.11 (1.51)	−0.44 (1.52)	0.91 (1.61)	10.68***	FALS versus HC	0.005
						SALS versus HC	<0.001
						FALS versus SALS	0.04

For parametric data, scores are reported as means (standard deviations), one-way ANOVA *F*-statistic and *post hoc* Bonferroni–Holm comparison *P*-value. ^a^Indicates data were not normally distributed and that scores are represented using medians (standard deviations), Kruskal–Wallis *H* statistic and *post hoc P*-values (comparisons that exceeded critical difference are marked as *P* < 0.05).

* *P* < 0.05; **P < 0.01; ***P < 0.001.

### Neuropsychiatric endophenotypes

Multiple linear regressions indicated that, after controlling for age, AQ attention to detail traits’ scores were significantly higher in ALS relatives than controls, *F*(2223) = 4.39, *P* = 0.01, *B* = 0.68, *P* = 0.01. In terms of personality traits, conscientiousness, *F*(2220) = 5.14, *P* = 0.007, *B* = −0.39, *P* = 0.008, and openness to experience, *F*(2220) = 4.83, *P* = 0.009, *B* = −0.47, *P* = 0.007, traits were both significantly lower in relatives of pwALS than controls, controlling for age. Summary statistics on neuropsychiatric trait scores in each group are provided in supplementary Table 2.

### Effect of family history on neuropsychiatric endophenotypes

**Figure 2:**
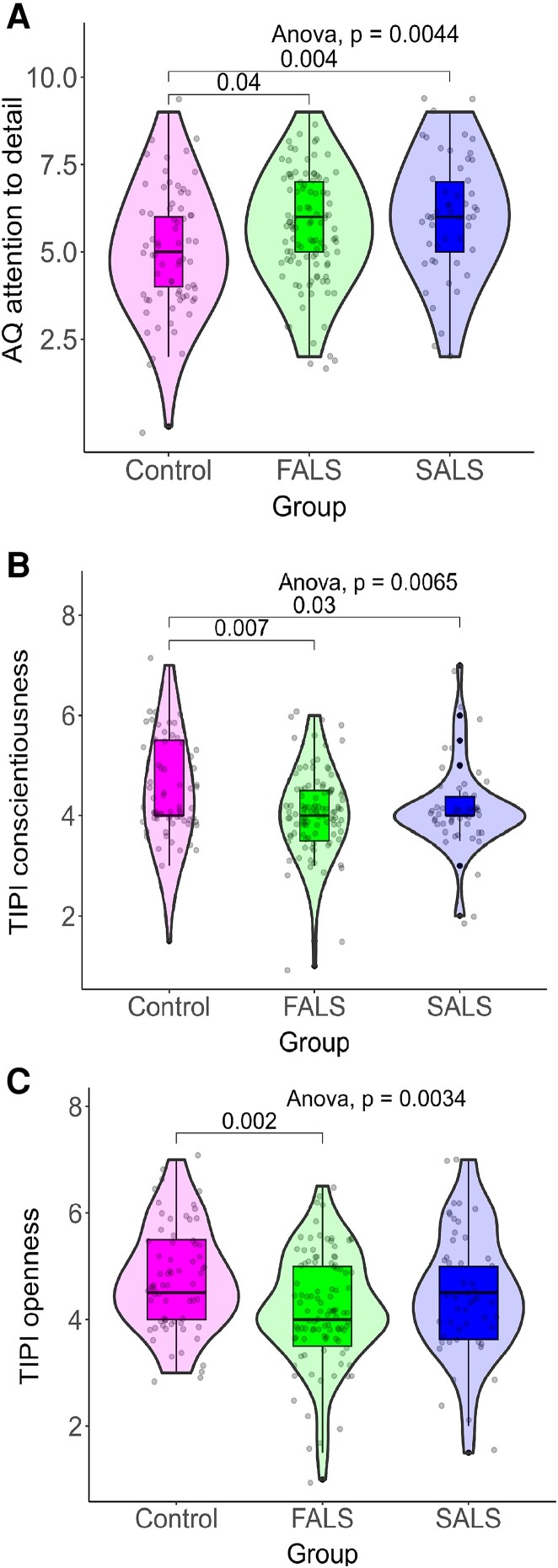
**Autism quotient attention to detail traits and Ten-Item Personality Inventory conscientiousness and openness to experience traits in FALS relatives, SALS relatives and controls. Violin plots of (A) AQ attention to detail traits and TIPI; (B) conscientiousness and (C) openness to experience traits in FALS relatives (green), SALS relatives (blue) and controls (red).** Each dot represents an individual data point from that group. Significant one-way ANOVA main effect on AQ attention to detail, *F*(2223) = 5.44, *P* = 0.0044. Tukey’s *post hoc* tests significant between FALS relatives and controls, *P* = 0.04, and between SALS relatives and controls, *P* = 0.004. Significant one-way ANOVA main effect on TIPI conscientiousness, *F*(2220) = 5.16, *P* = 0.0085. Tukey’s *post hoc* tests significant between FALS relatives and controls, *P* = 0.007, and between SALS relatives and controls, *P* = 0.03. Significant one-way ANOVA main effect on TIPI openness to experience, *F*(2220) = 5.82, *P* = 0.0034. Tukey’s *post hoc* tests significant between FALS relatives and controls, *P* = 0.002. FALS = familial amyotrophic lateral sclerosis; SALS = sporadic amyotrophic lateral sclerosis; AQ = autism quotient; TIPI = Ten-Item Personality Inventory.

Multiple linear regressions were also carried out to determine whether family history (i.e. FALS relative, SALS relative, control) was associated with neuropsychiatric traits scores, while controlling for the effect of age. SALS relatives had significantly higher AQ attention to detail traits than controls, *F*(3222) = 3.75, *P* = 0.01, *B* = 0.95, *P* = 0.004). Both FALS (*B* = −0.4, *P* = 0.01) and SALS (*B* = −0.36, *P* = 0.03) relatives had significantly lower conscientiousness traits than controls, *F*(3219) = 3.43, *P* = 0.02). For openness to experience, only FALS relatives (*B* = −0.57, *P* = 0.003) scored significantly lower than controls, *F*(3219) = 3.88, *P* = 0.01 (see Fig. 2).

## Discussion

These findings suggest that relatives of ALS probands, both *C9orf72* positive and negative, exhibit poorer phonemic verbal fluency and object naming when compared to healthy controls with no family history of ALS. Phonemic fluency deficits are well established as one of the most sensitive markers of impairment in pwALS and have been associated with impaired activation of middle and inferior frontal gyri, the anterior cingulate gyrus, and reduced activity in parietal and temporal lobes.^43^ Abnormal verbal fluency has also previously been observed in asymptomatic *C9orf72* gene carriers;^38,39^ however, this study indicates that verbal fluency deficits may also present in *C9orf72* negative family members (see Fig. 1B).

The effect size of the phonemic verbal fluency deficit was large and presented specifically in relatives of people with familial ALS, rather than relatives of people with sporadic ALS, suggesting it may segregate with those at higher risk of ALS, a defining feature of a disease endophenotype.^44^ Notably, a similar pattern of verbal fluency deficits (in this case, semantic fluency) has been observed in unaffected siblings and parents of people with schizophrenia,^45,46^ whereby relatives of people with familial schizophrenia had significantly poorer semantic verbal fluency than relatives of people with sporadic schizophrenia.

Apart from phonemic verbal fluency, relatives of pwALS performed worse than controls on object naming, inhibition, working memory, immediate list learning and visuospatial memory and delayed story recall. However, rather than viewing each findings in isolation, it is likely that the deficits observed in multiple cognitive processes are attributable to dysfunction of brain networks and cognitive functions that are fundamental to multiple processes, such as the fronto-striatal networks responsible for the various executive functions, or the frontotemporal networks more associated with language.^47^ ALS is increasingly recognized as a network disorder,^5^ which may manifest across numerous cognitive tests. A similar, albeit less pronounced dysfunction in relatives, may underlie the cognitive dysfunction observed in this sample and may be indicative of the possible neurodevelopmental nature of the disease. It is also important to note that these observed deficits are subclinical at a group level, indicating subtle traits, with few individuals scoring below clinical thresholds (e.g. >2 SD below control mean performance).

Relatives of those with familial ALS reported greater levels of ASD traits. This finding was specific to a subset of ASD traits relating to attention to detail, which refers to the extent to which individuals attend to fine grain details at the expense of more integrative perceptions.^48^ The use of this specific trait may be a more promising marker of the association between ALS and autism than a more global measure of ASD. Greater attention to detail ASD traits are also likely reflected in the personality characteristics observed in relatives, i.e. low scores on openness to experience and conscientiousness. While data pertaining to personality characteristics in pwALS is limited, some studies suggest that ALS patients are more likely to display a high degree of emotional control^49^ and lower openness to experience scores than patients with other chronic, progressive diseases, such as multiple sclerosis and cancer.^40^

### Limitations

ALS relatives were recruited on a voluntary basis, and those with active mental illness or a history of alcohol abuse were excluded, resulting in a selection bias. If relatives were experiencing symptoms or psychotic disorders, such as social isolation or withdrawal from society, this meant that they would not have participated due to these symptoms.^50^ Selection bias may also have resulted from the differing recruitment strategies for cases and controls, i.e. snowball sampling versus random population sampling. The exclusion of individuals with learning or developmental disabilities may have further obscured the identification of cognitive endophenotypes, particularly in families with the *C9orf72* repeat expansion, where ALS is increasingly viewed as a neurodevelopmental disorder.^38^ This is possibly reflected in the relatively low sampling of *C9orf72* positive family members from *C9orf72* families (*n* = 16/44, 36%).

While the utilization of age-specific norms reduced the confounding effect of age for most tests, these norms were typically developed in American populations and may not be optimal for an Irish cohort. The neuropsychological assessment was largely tailored towards executive functioning, due to its relevance in ALS. However, object naming was one of the most impaired functions in unaffected relatives. A more detailed language assessment may reveal further insight into possible language-related endophenotypes. Despite the large sample of ALS relatives, relatively few were *C9orf72* positive, limiting the power to detect gene-related differences. While researchers were blinded to *C9orf72* status, they were not blinded to case status (i.e. control or family member) potentially resulting in interviewer bias. Lastly, this study is limited by only testing for the *C9orf72* repeat expansion, as, with the exception of one kindred carrying a FUS mutation that was not included in this study, other known ALS-associated genes have not been detected in the Irish population.

While this study has primarily emphasized genetic factors which may influence cognitive and neuropsychiatric outcomes, it is important to consider additional psychosocial factors which may mediate and/or moderate these relationships. While our sample of ALS relatives matched controls in terms of education, they may have differed or other socioeconomic indicators, such as parental education and parental occupation.

## Conclusions

Notwithstanding these limitations, our data suggest that first- and second-degree relatives of pwALS display differences on cognitive functioning and neuropsychiatric traits, which may constitute a disease endophenotype. Both *C9orf72* gene positive and non-*C9orf72* relatives exhibited lower phonemic verbal fluency, object naming and backwards digit span and exhibited higher ASD traits, pointing to the presence of a disease-associated endophenotype that is not exclusively mediated by the *C9orf72* expansion. These observations demonstrate that relatives of ALS probands should not be used as controls in future studies, as factors, possibly genetic, that drive susceptibility to ALS can be detected within extended kindreds.

## Supplementary Material

fcad166_Supplementary_Data

## Data Availability

The data that support the findings of this study are available on request from the corresponding author. The data are not publicly available due to their containing information that could compromise the privacy of research participants.

## Abbreviations

ALS =: amyotrophic lateral sclerosis
AQ =: autism spectrum quotient
ASRS =: Adult ADHD Self-Report Scale
BIS =: Barrett Impulsiveness Scale
BNT =: Boston Naming Task
CAPE =: Community Assessment of Psychic Experiences
CWIT =: Colour Word Interference Task
DAS =: Dimensional Apathy Scale
FALS =: familial amyotrophic lateral sclerosis
FTD =: frontotemporal dementia
FTSD =: frontotemporal spectrum disorder
GAD =: Generalized Anxiety Disorder
IGT =: Iowa Gambling Task
OCD =: obsessive compulsive disorder
OCI-R =: Obsessive Compulsive Inventory Revised
PHQ =: Patient Health Questionnaire
pwALS =: people with ALS
RAVLT =: Rey Auditory Verbal Learning Test
RCFT =: Rey Complex Figure Test
RMET =: Reading the Mind in the Eyes Task
SALS =: sporadic amyotrophic lateral sclerosis
TIPI =: Ten-Item Personality Inventory
TOPF-UK =: Test of Premorbid Functioning UK
WASI-II =: Wechsler Abbreviated Scale of Intelligence 2nd edition
WMS-III =: Wechsler Memory Scale—3rd edition (WMS-III)
