# Supplementary material for: Cognitive and neuropsychiatric endophenotypes in amyotrophic lateral sclerosis
Source: Brain Commun. 2023 May 19;5(3):fcad166. doi: 10.1093/braincomms/fcad166 (PMC10243911; doi:10.1093/braincomms/fcad166)
Supplement: fcad166_Supplementary_Data [file fcad166_supplementary_data.pdf]

Supplemental table 1. Bonferroni-Holm adjusted alpha level for each statistical test.

		t/W (df)	*P*	*Adjusted α*
TOPF-UK	*FSIQ*	3.02 (106)	.003	.002
WASI-II	*FSIQ-2*	2.62 (94)	.01	.003
Verbal fluency	*FAS total z*	3.79 (89)	.0003	.0019
	Animals total z	0.58 (94)	.56	.01
Colour-word interference test	*Inhibition errors ss*	3.08 (158)	.002	.0024
	Inhibition time ss	1.66 (120)	.10	.004
	Switching errors ss	0.73 (106)	.47	.007
	Switching time ss	1.76 (106)	.08	.003
Digit span	*Forward z*[a]	4735	.51	.01
	Backwards z	4.19 (109)	.00006	.0017
	Sequential z	0.56 (115)	.57	.03
Iowa Gambling Task	*Total t*	2.36 (92)	.02	.003
	Block 1 t	-0.97 (87)	.34	.006
	Block 2 t	0.7 (85)	.48	.008
	Block 3 t	1.14 (87)	.26	.005
	Block 4 t	1.68 (77)	.1	.004
	Block 5 t	2.93 (97)	.004	.002
BNT	*Spontaneous z*	6.42 (201)	<.00001	.0017
	Cued z	6.01 (201)	<.00001	.0017
RAVLT	*Immediate z*	3.31 (98)	.001	.002
	Delayed z	2.52 (88)	.01	.003
	Recognition z[a]	3661	.38	.006
Logical Memory	*Immediate z*	-1.17 (108)	.25	.005
	Delayed z	3.48 (98)	.0007	.0019
	Recognition raw	1.2 (128)	.23	.004
RCFT	*Copy z*[a]	4003.5	.94	.05
	Copy time z	-3.36 (144)	.001	.002
	Immediate z	3.99 (91)	.0001	.0018
	Delayed z[a]	4843	.005	.002
	Recognition z	2.85 (118)	.005	.002
RMET	*Total z*	0.13 (136)	.9	.003

Supplemental Table 2. Comparison of neuropsychiatric traits of ALS relatives and controls.

	**Relative (n=147)**	**Controls (n=60)**
PHQ total[a]	1 (3.14)	2 (2.24)
GAD total[a]	2 (4.32)	0 (5.07)
CAPE-P15 total[a]	1 (2.17)	1 (1.47)
OCIR total[a]	5 (5.85)	5 (5.47)
AQ total	22.42 (4.74)	22.97(4.6)
AQ social skill	3.62 (1.31)	3.42 (1.29)
AQ attention switching	3.8 (1.65)	3.87 (1.57)
AQ attention to detail	5.75 (1.62)	5.04 (1.74)
AQ communication	5.45 (1.49)	5.55 (1.5)
AQ imagination	4.34 (1.4)	4.54 (1.62)
DAS total	25.36 (7.04)	25.36 (7.15)
DAS executive[a]	6 (4.13)	7 (4.11)
DAS emotional	7.79 (3.26)	8.48 (3.18)
DAS initiation	11.39 (3.64)	10.05 (3.6)
ARSR total[a]	1 (1.53)	1 (1.32)
BIS total	55.5 (8.58)	54.59 (8.28)
TIPI extraversion	4.42 (0.98)	4.62 (0.96)
TIPI agreeableness	4.61 (0.93)	4.46 (0.89)
TIPI conscientiousness	4.09 (0.81)	4.49 (0.95)
TIPI emotional stability	4.7 (0.95)	4.59 (0.85)
TIPI openness to experience	4.3 (1.08)	4.78 (0.97)

For parametric data, scores are reported as means (standard deviations), [a] indicates data were not normally distributed and that scores are reported using medians (standard deviations),

Supplemental table 3. Hierarchical multiple regression summary, predicting cognitive performance from WASI-II FSIQ-2 and group status (i.e., relative of pwALS vs control; n = 209).

**Outcome = FAS**	**ΔR^2**	**B**	**SE B**	**β**	**p**
Step 1	0.19				<.001
Constant		-3.5	0.5		<.001
FSIQ-2		.03	0.005	0.44	<.001
Step 2	0.03				.005
Constant		-2.89	0.53		<.001
FSIQ-2		.03	0.005	0.40	<.001
Group		-0.47	0.17	-.18	.005
**Outcome = Digit span backwards**	**ΔR^2**	**B**	**SE B**	**β**	**p**
Step 1	0.16				<.001
Constant		-2.52	0.46		<.001
FSIQ-2		.03	0.005	0.40	<.001
Step 2	0.04				.004
Constant		-1.96	0.49		<.001
FSIQ-2		.03	0.004	0.37	<.001
Group		-0.43	0.15	-.19	.004
Outcome = **CWIT inhibition errors**	**ΔR^2**	**B**	**SE B**	**β**	**p**
Step 1	0.15				<.001
Constant		-1.9	0.37		<.001
FSIQ-2		.02	0.004	0.39	<.001
Step 2	0.01				.15
Constant		-1.7	0.4		<.001
FSIQ-2		.02	0.004	0.37	<.001
Group		-0.18	0.12	-0.1	.15
**Outcome = BNT spontaneous**	**ΔR2**	**B**	**SE B**	**β**	**p**
Step 1	0.1				<.001
Constant		-5.22	0.83		<.001
FSIQ-2		0.04	0.008	0.32	<.001
Step 2	0.06				<.001
Constant		-3.98	0.87		<.001
FSIQ-2		0.03	0.008	0.27	<.001
Group		-0.97	0.26	-0.24	<.001
**Outcome = RAVLT immediate recall**	**ΔR2**	**B**	**SE B**	**β**	**p**
Step 1	0.23				<.001
Constant		-3.46	0.54		<.001
FSIQ-2		0.04	0.005	0.48	<.001
Step 2	0.01				.06
Constant		-3.02	0.58		<.001
FSIQ-2		0.04	0.005	0.46	<.001
Group		-0.33	0.18	-0.12	.06

**Outcome = LM delayed recall**	**ΔR2**	**B**	**SE B**	**β**	**p**
Step 1	0.21				<.001
Constant		0.9	1.44		.53
FSIQ-2		0.1	0.01	0.46	<.001
Step 2	0.03				.06
Constant		1.99	1.56		<.001
FSIQ-2		0.09	0.01	0.44	<.001
Group		-0.83	0.47	-0.12	.08
**Outcome = RCFT Immediate recall**	**ΔR2**	**B**	**SE B**	**β**	**p**
Step 1	0.07				<.001
Constant		-4.02	1.08		<.001
FSIQ-2		0.4	0.01	0.27	<.001
Step 2	0.003				.52
Constant		-3.66	1.21		.003
FSIQ-2		0.04	0.01	0.26	<.001
Group		-0.24	0.37	-0.05	.52
